# Trends in urolithiasis mortality in the United States, 1999–2024: a Joinpoint regression analysis of age, sex, race, and regional disparities

**DOI:** 10.3389/fsurg.2026.1853819

**Published:** 2026-08-21

**Authors:** Li-Li Xu, Chu-xuan Lin, Xin-yi Lin, Qin-ying Zhou, Nai-fen Xu, Ding-qin Zheng, Ran Xu

**Affiliations:** Department of Urology, Pingyang Hospital of Wenzhou Medical University, Wenzhou, China

**Keywords:** age-adjusted mortality rates, CDC WONDER, epidemiology, mortality, urolithiasis

## Abstract

**Background:**

Urolithiasis is traditionally considered a benign condition with extremely low mortality. However, recent evidence suggests a potential shift in this paradigm. This study aims to analyze urolithiasis mortality trends in the United States from 1999 to 2024, with a focus on demographic and geographic disparities.

**Methods:**

Death certificate data for individuals aged 45 years and older were obtained from the CDC WONDER database. Urolithiasis-related deaths were identified using ICD-10 codes N20–N23. Age-adjusted mortality rates (AAMRs) were calculated, and Joinpoint regression was employed to identify significant changes in mortality trends. Analyses were stratified by census region, sex, race/ethnicity, and age group.

**Result:**

From 1999 to 2024, the age-adjusted mortality rate for urolithiasis increased from 0.22 to 0.72 per 100,000 population. The rise was modest from 1999 to 2014, with an annual percent change of 1.94%, followed by a sharp acceleration from 2014 to 2024, when the annual percent change reached 8.54%. Substantial disparities were observed. The Northeast had the highest age-adjusted mortality rate at 0.85 per 100,000, while the Midwest experienced the fastest increase, with an average annual percent change of 5.42%. In 2024, the female age-adjusted mortality rate surpassed that of males (0.80 vs. 0.68 per 100,000). Non-Hispanic white people bore the heaviest burden, with an age-adjusted mortality rate of 0.86 per 100,000 and an average annual percent change of 5.71%. Hispanics exhibited a “mortality paradox,” whereas non-Hispanic black people had the lowest rate at 0.36 per 100,000. Mortality increased with age: the highest rate was observed among those aged 85 years and older (5.72 per 100,000), and the fastest increase occurred in the 65–74 years age group, with an average annual percent change of 4.94%.

**Conclusion:**

Urolithiasis mortality in the United States has increased dramatically since 2014, with persistent disparities observed across region, sex, race, and age. These findings suggest that urolithiasis mortality in the United States has increased substantially since 2014, warranting increased public health attention. However, given the low absolute mortality rates and the inherent limitations of death certificate data, these findings should be interpreted as hypothesis-generating and require confirmation in future studies. Targeted interventions are needed for high-risk groups—including older adults, females, non-Hispanic white people, and individuals with metabolic syndrome—as well as for high-risk regions such as the Midwest and rural areas.

## 1 Introduction

Urolithiasis is one of the most common diseases of the urinary tract and can be classified as upper or lower urinary tract stones depending on the location. Epidemiological studies have shown that both the prevalence and mortality of urolithiasis are increasing worldwide (1). Moreover, due to differences in social conditions, dietary habits, climate, and other factors across continents and countries, significant variations in prevalence and mortality exist among different regions and racial/ethnic groups (2).

Urolithiasis can lead to a range of clinical symptoms, including urinary tract infection, hematuria, and pain. In severe cases, it may progress to urosepsis and septic shock (3). The clinical manifestations and worsening epidemiological profile of urolithiasis have placed a substantial burden on global public health. One study estimated that the annual cost of treating urolithiasis in the United States was $3.79 billion in 2007, with an expected average annual increase of $1.24 billion through 2030 (4).

In response to this challenge, a systematic analysis of statistically rigorous and representative large-scale datasets is necessary to provide actionable insights for policymakers and public health professionals, thereby facilitating the development of targeted interventions. Therefore, this study leverages the CDC WONDER database to conduct a multi-dimensional analysis of urolithiasis mortality in the United States from 1999 to 2024.

## 2 Methods

### 2.1 Data resources

CDC WONDER (https://wonder.cdc.gov/) is a public health data and communication system developed by the Centers for Disease Control and Prevention. This database comprehensively contains officially recorded death counts and causes of death for all regions of the United States from 1999 to 2024, and has been widely recognized and extensively used by public health professionals (5, 6).

### 2.2 Definition of study population and variables

This study focused on urolithiasis-related deaths among adults aged 45 years and older over the 26-year period from 1999 to 2024. In the CDC WONDER database, urolithiasis cases were identified and collected using the International Classification of Diseases, Tenth Revision (ICD-10) codes N20–N23 as the underlying cause of death. The CDC WONDER Underlying Cause of Death database attributes each death to a single underlying cause, which is defined as the disease or injury that initiated the chain of events leading directly to death. Furthermore, the CDC WONDER database contains only de-identified data and adheres to the STROBE (Strengthening the Reporting of Observational Studies in Epidemiology) reporting guidelines (7); therefore, institutional review board approval was not required.

We extracted multiple variables from the CDC WONDER database, including demographic parameters (sex, race/ethnicity, and age) and geographic location. Race/ethnicity categories included Hispanic or Latino, Black or African American, and White individuals. Age was stratified into the following 10-year groups: 45–54 years, 55–64 years, 65–74 years, 75–84 years, and 85 years and older. Geographic variables were primarily based on census regions, as defined by the U.S. Census Bureau into four major regions: Northeast, Midwest, South, and West.

### 2.3 Statistical analysis

Throughout the study, we comprehensively analyzed the absolute number of deaths, crude mortality rates, and age-adjusted mortality rates (AAMRs). To more rigorously assess changes in mortality trends over time, we performed Joinpoint regression analysis. The Joinpoint regression model uses a rigorous Monte Carlo permutation test to identify statistically significant inflection points in temporal mortality trends, thereby effectively partitioning the entire time series into contiguous linear segments (8–10). Model selection was performed using the Weighted Bayesian Information Criterion (WBIC), with a maximum of 4 joinpoints allowed based on the 26 annual data points in our series. The permutation test was conducted with 4,500 permutations, consistent with the default settings in Joinpoint version 5.1.0.0. We then calculated the annual percent change (APC) for each segment, the average annual percent change (AAPC) for the overall trend, and their respective 95% confidence intervals. Statistical significance was set at *P* < 0.05.

All analyses were primarily conducted using R software (version 4.2.3) and Joinpoint software (version 5.1.0.0).

## 3 Results

### 3.1 Overall mortality trends

At the national level, urolithiasis mortality trends worsened dramatically (Table 1 and Supplementary Table S1). Specifically, the number of urolithiasis-related deaths increased from 227 in 1999 to 1,128 in 2024, and the AAMR rose from 0.22 per 100,000 population (95% CI: 0.19 to 0.25) in 1999 to 0.72 per 100,000 population (95% CI: 0.68 to 0.76) in 2024.

**Table 1:** Trends in urolithiasis mortality in the United States, 1999–2024.

Characteristic	Deaths		AAMR^a^		
	1999	2024	1999 (95% CI)	2024 (95% CI)	AAPC (95% CI)
USA	227	1128	0.22 (0.19 to 0.25)	0.72 (0.68 to 0.76)	4.53 (3.64 to 5.43)
Census Region					
Midwest	58	239	0.27 (0.20 to 0.35)	0.72 (0.63 to 0.82)	5.42 (3.88 to 6.98)
Northeast	44	247	0.21 (0.15 to 0.29)	0.85 (0.75 to 0.97)	5.01 (3.94 to 6.09)
South	71	377	0.21 (0.16 to 0.27)	0.66 (0.60 to 0.73)	4.41 (3.44 to 5.39)
West	54	265	0.27 (0.20 to 0.36)	0.77 (0.68 to 0.87)	4.33 (3.12 to 5.56)
Sex					
Female	136	685	0.22 (0.18 to 0.25)	0.80 (0.74 to 0.86)	4.91 (4.20 to 5.63)
Male	91	443	0.23 (0.18 to 0.29)	0.68 (0.62 to 0.75)	4.60 (3.60 to 5.06)
Race					
Hispanic	20	63	0.47 (0.28 to 0.75)	0.37 (0.28 to 0.48)	−1.45 (−5.14 to 2.39)
NH Black	19	56	0.27 (0.15 to 0.39)	0.36 (0.27 to 0.48)	1.52 (−0.81 to 3.91)
NH White	183	973	0.22 (0.18 to 0.25)	0.86 (0.81 to 0.92)	5.71 (4.96 to 6.46)
Age Groups					
45–54 years	10	33	0.03 (0.01 to 0.05)	0.08 (0.06 to 0.11)	3.10 (0.98 to 5.26)
55–64 years	30	103	0.13 (0.09 to 0.18)	0.25 (0.20 to 0.29)	3.62 (2.01 to 5.26)
65–74 years	40	252	0.22 (0.16 to 0.30)	0.71 (0.62 to 0.80)	4.94 (3.67 to 6.22)
75–84 years	69	372	0.56 (0.44 to 0.71)	1.93 (1.73 to 2.12)	4.50 (3.44 to 5.58)
85 + years	78	368	1.88 (1.48 to 2.84)	5.72 (5.13 to 6.30)	4.90 (3.81 to 6.00)

AAPC, average annual percent change presented for full period; CI, confidence interval; AAMR, age-adjusted mortality rate.

a For the age groups, the crude mortality rate was used as a substitute for AAMR, and the AAPC was computed based on the crude mortality rate.

**Figure 1:**
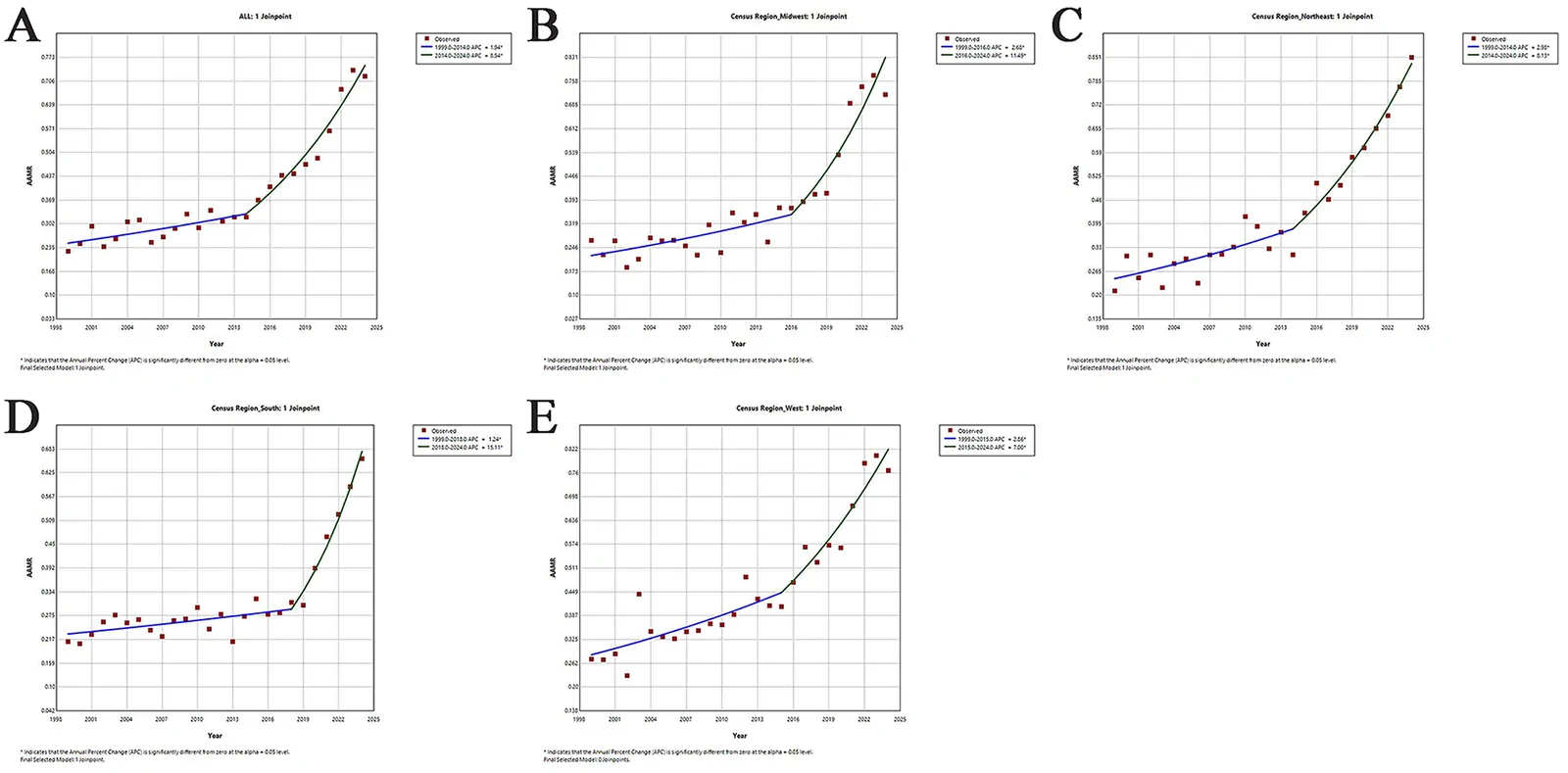
Joinpoint regression analysis of urolithiasis mortality in the United States and by census region, 1999–2024. **(A)** USA; **(B)** Midwest; **(C)** Northeast; **(D)** South; **(E)** West.

According to the Joinpoint regression analysis (Figure 1 and Table 2), the AAMR for urolithiasis in the United States first increased modestly from 1999 to 2014 (APC: 1.94%; 95% CI: 0.74% to 3.16%), followed by a sharp acceleration from 2014 to 2024 (APC: 8.54%; 95% CI: 7.02% to 10.08%). Both trends were statistically significant.

**Table 2:** Joinpoint regression analysis of urolithiasis mortality trends in the United States, 1999–2024.

	Year	APC/AAPC (%)	95%CI	Test Statistic (*t*)	*P*-Value
USA	1999-2014	1.943	0.744 to 3.156	3.382	0.003
	2014-2024	8.541	7.023 to 10.081	12.103	< 0.001
	1999-2024	4.532	3.644 to 5.429	10.173	< 0.001
Midwest	1999-2016	2.675	0.978 to 4.401	3.294	0.003
	2016–2024	11.490	7.813 to 15.293	6.744	< 0.001
	1999–2024	5.417	3.878 to 6.980	7.028	< 0.001
Northeast	1999–2014	2.979	1.520 to 4.458	4.280	< 0.001
	2014–2024	8.131	6.342 to 9.951	9.741	< 0.001
	1999–2024	5.010	3.941 to 6.089	9.366	< 0.001
South	1999–2018	1.242	0.371 to 2.120	2.972	0.007
	2018–2024	15.114	11.602 to 18.737	9.447	< 0.001
	1999–2024	4.410	3.439 to 5.391	9.050	< 0.001
West	1999–2015	2.862	1.351 to 4.395	3.966	0.001
	2015–2024	6.999	4.640 to 9.412	6.309	< 0.001
	1999–2024	4.332	3.119 to 5.560	7.105	< 0.001
Female	1999–2015	2.782	1.899 to 3.672	6.618	< 0.001
	2015–2024	8.812	7.410 to 10.232	13.542	< 0.001
	1999–2024	4.913	4.201 to 5.630	13.799	< 0.001
Male	1999–2016	1.616	0.504 to 2.740	3.031	0.006
	2016–2024	11.218	8.840 to 13.649	10.230	< 0.001
	1999–2024	4.595	3.596 to 5.604	9.170	< 0.001
Hispanic	1999–2004	−17.061	−31.280 to 0.101	−2.069	0.051
	2004–2024	2.895	0.986 to 4.841	3.168	0.005
	1999–2024	−1.447	−5.137 to 2.386	−0.749	0.454
NH Black	1999–2011	−3.171	−7.138 to 0.966	−1.602	0.124
	2011–2024	6.058	3.171 to 9.026	4.432	< 0.001
	1999–2024	1.523	−0.811 to 3.912	1.274	0.203
NH White	1999–2017	3.774	3.020 to 4.534	10.564	< 0.001
	2017–2024	10.840	8.738 to 12.982	11.180	< 0.001
	1999–2024	5.706	4.961 to 6.456	15.379	< 0.001
45–54 years	1999–2018	1.868	0.218 to 3.546	2.358	0.028
	2018–2024	7.082	−0.724 to 15.502	1.880	0.074
	1999–2024	3.096	0.980 to 5.256	2.882	0.004
55–64 years	1999–2014	1.812	−0.319 to 3.989	1.766	0.092
	2014–2024	6.392	3.578 to 9.283	4.807	< 0.001
	1999–2024	3.620	2.010 to 5.256	4.451	< 0.001
65–74 years	1999–2016	2.687	1.225 to 4.170	3.846	0.001
	2016–2024	9.883	7.006 to 12.837	7.388	< 0.001
	1999–2024	4.937	3.666 to 6.223	7.752	< 0.001
75–84 years	1999–2014	1.879	0.447 to 3.331	2.734	0.012
	2014–2024	8.567	6.757 to 10.408	10.167	< 0.001
	1999–2024	4.503	3.441 to 5.576	8.455	< 0.001
85 + years	1999–2016	1.998	0.801 to 3.209	3.486	0.002
	2016–2024	11.332	8.703 to 14.025	9.342	< 0.001
	1999–2024	4.896	3.806 to 5.998	8.968	< 0.001

AAPC, average annual percent change presented for full period; APC, annual percent change; CI, confidence interval.

### 3.2 Census region

From 1999 to 2024, urolithiasis mortality trends were similar across different census regions (Table 1 and Supplementary Table S1). Among the four census regions, the Midwest had the fewest deaths in 2024 (239 cases) but the fastest increase in AAMR, with an AAPC of 5.42% (95% CI: 3.88% to 6.98%). The Northeast, despite having only 44 deaths in 2024—the second lowest after the Midwest—had the highest AAMR among the four regions, at 0.85 per 100,000 population (95% CI: 0.75 to 0.97). The South had the highest number of deaths (377 cases) in 2024 but the lowest AAMR, at 0.66 per 100,000 population (95% CI: 0.60 to 0.73). Finally, the West had the slowest increase in AAMR, with an AAPC of 4.33% (95% CI: 3.12% to 5.56%).

According to the Joinpoint regression analysis (Figure 1 and Table 2), all four census regions exhibited a similar mortality trend characterized by a modest increase followed by a sharp acceleration. The only notable difference was the timing of the inflection point across regions.

### 3.3 Sex

The number of urolithiasis-related deaths increased from 136 in 1999 to 685 in 2024 among females, and from 91 in 1999 to 443 in 2024 among males. Female AAMR rose from 0.22 per 100,000 population (95% CI: 0.18 to 0.25) in 1999 to 0.80 per 100,000 population (95% CI: 0.74 to 0.86) in 2024, while male AAMR rose from 0.23 per 100,000 population (95% CI: 0.18 to 0.29) in 1999 to 0.68 per 100,000 population (95% CI: 0.62 to 0.75) in 2024.

**Figure 2:**
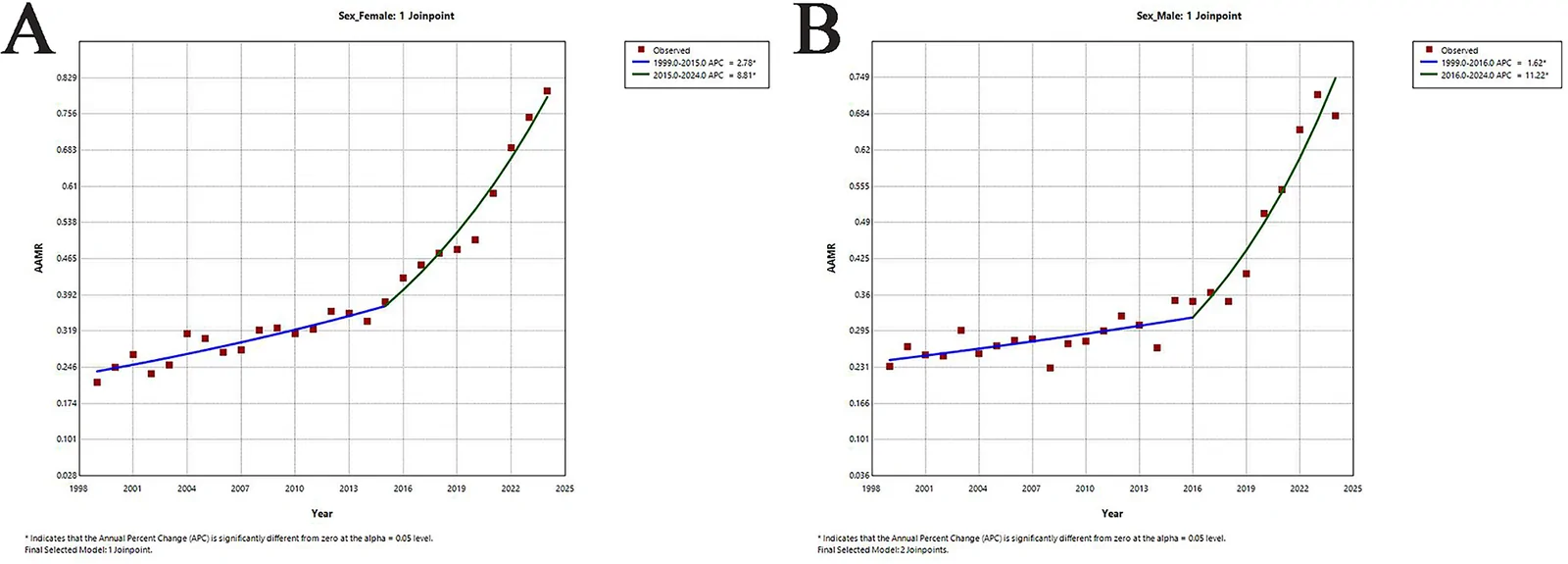
Joinpoint regression analysis of urolithiasis mortality by sex in the United States, 1999–2024. **(A)** Female; **(B)** Male.

According to the Joinpoint regression analysis (Figure 2 and Table 2), both sexes exhibited a similar mortality trend characterized by a modest increase followed by a sharp acceleration. The sharp acceleration occurred from 2015 to 2024 among females and from 2016 to 2024 among males.

### 3.4 Race and ethnicity

Compared with sex, urolithiasis mortality disparities were more pronounced across racial and ethnic groups. Non-Hispanic white people had the highest mortality burden. Specifically, among non-Hispanic white people, the number of urolithiasis-related deaths increased from 183 in 1999 to 973 in 2024, and the AAMR rose from 0.22 per 100,000 population (95% CI: 0.18 to 0.25) in 1999 to 0.86 per 100,000 population (95% CI: 0.81 to 0.92) in 2024. Moreover, the AAPC for non-Hispanic white people was also very high, at 5.71% (95% CI: 4.96% to 6.46%). In contrast, the AAMR for Hispanics decreased from 0.47 per 100,000 population (95% CI: 0.28 to 0.75) in 1999 to 0.37 per 100,000 population (95% CI: 0.28 to 0.48) in 2024. Finally, compared with other racial/ethnic groups, non-Hispanic black people had the lowest AAMR in 2024, at 0.36 per 100,000 population (95% CI: 0.15 to 0.39).

**Figure 3:**
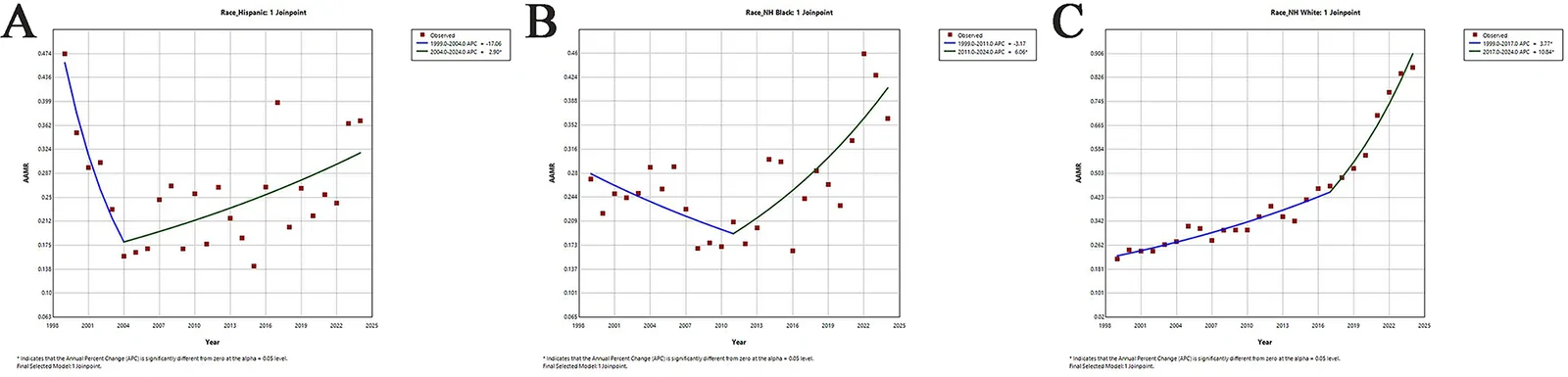
Joinpoint regression analysis of urolithiasis mortality by race and ethnicity in the United States, 1999–2024. **(A)** Hispanic; **(B)** NH Black; **(C)** NH White.

Similarly, marked differences were observed in Joinpoint regression analysis across racial and ethnic groups (Figure 3 and Table 2). Hispanics and non-Hispanic black people exhibited a trend characterized by an initial decrease followed by a subsequent increase, whereas non-Hispanic white people showed a modest increase followed by a sharp acceleration. Notably, only the APC and AAPC for non-Hispanic white people reached statistical significance.

### 3.5 Age group

Analysis of urolithiasis mortality by age group (Table 1) revealed that as age increased, the number of deaths first rose and then slightly declined, with the 75–84 years age group having the highest number of deaths (372 cases in 2024). In contrast, mortality rates showed a consistent increasing trend with age, reaching the highest level among those aged 85 years and older, at 5.72 per 100,000 population (95% CI: 5.13 to 6.30) in 2024.

**Figure 4:**
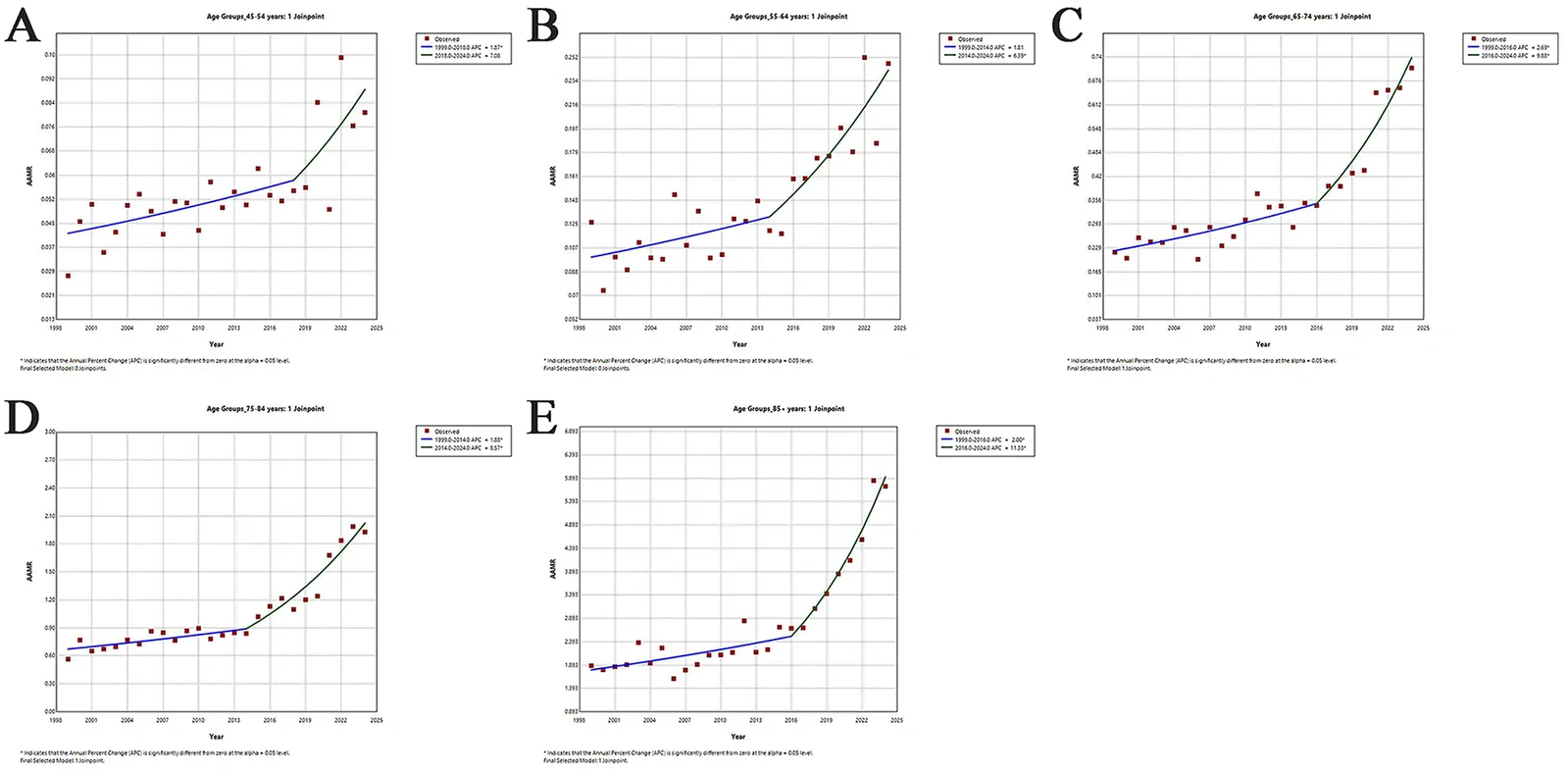
Joinpoint regression analysis of urolithiasis mortality by age group in the United States, 1999–2024. **(A)** 45-54; **(B)** 55-64; **(C)** 65-74; **(D)** 75-84; **(E)** 85 + .

According to the Joinpoint regression analysis (Figure 4 and Table 2), all age groups exhibited a similar mortality trend characterized by a modest increase followed by a sharp acceleration. The 65–74 years age group had the highest AAPC among all age groups, at 4.94% (95% CI: 3.67% to 6.22%). Finally, only the 45–54 years age group showed an APC from 2018 to 2024 that did not reach statistical significance.

## 4 Discussion

This study found that from 1999 to 2024, AAMR for urolithiasis in the United States followed a two-phase upward trend—initially modest, then sharp—increasing from 0.22 to 0.72 per 100,000 population, more than tripling over the 25-year period. The increase accelerated dramatically after 2014, reaching an annual rate of 8.54% between 2014 and 2024. By census region, the Northeast had the highest AAMR at 0.85 per 100,000 population, while the Midwest experienced the fastest increase with an average annual percent change of 5.42%. The South had the highest number of deaths but the lowest AAMR. The timing of the inflection point showed a spatial gradient, occurring earliest in the Northeast and latest in the South. By sex, female AAMR surpassed that of males, and the inflection point occurred earlier in females than in males. By race/ethnicity, non-Hispanic white people bore the heaviest burden, with AAMR rising from 0.22 to 0.86 per 100,000 and an AAPC of 5.71%; Hispanics exhibited lower mortality rates with a declining trend; non-Hispanic black people had the lowest AAMR. By age group, the highest mortality rate was observed among those aged 85 years and older (5.72 per 100,000), while the fastest increase occurred in the 65–74 years age group (AAPC: 4.94%). Although the 45–54 years age group showed a late-period APC of 7.08%, it did not reach statistical significance. These findings suggest that urolithiasis mortality has increased substantially in recent years, particularly among certain demographic subgroups. However, given the low absolute mortality rates and the inherent limitations of death certificate-based data, these observations should be interpreted as hypothesis-generating rather than conclusive, and warrant further investigation in future studies.

At the national level, the AAMR for urolithiasis in the United States showed a significant and continuously worsening trend. Previous studies have largely focused on the prevalence and incidence of urolithiasis, with relatively outdated data on mortality. Sammon et al. previously suggested that urolithiasis mortality had long remained extremely low and stable (11). However, our findings contradict this view, demonstrating a notable change over the past decade—such a sharp national increase has rarely been reported before. Notably, entering the mid-2010s, the mortality burden of urolithiasis in the United States began to accelerate at a substantially faster pace. This temporal turning point represents an important but unexplained structural change in mortality trends that warrants further investigation. Several hypotheses may plausibly contribute to this acceleration, though they cannot be directly tested with the current dataset.

First, the rising prevalence of obesity and diabetes may have exerted a delayed effect on severe stone complications (12, 13). However, given that obesity trends evolved gradually rather than abruptly, this mechanism alone does not clearly explain the sharp inflection observed after 2014. Second, the mid-2010s saw the rapid community spread of fluoroquinolone-resistant *E. coli* in the United States (14). Patients with urolithiasis are at increased risk for such infections, and their temporal overlap with the mortality acceleration is noteworthy, though this association remains ecological and non-causal (15).

Significant disparities were observed across census regions. The higher AAMR in the Northeast may be attributed to its highly aging population and the greater burden of comorbidities among older adults (16–18). The Midwest had the highest AAPC, which may be related to its higher proportion of rural residents and the sparse distribution of specialized stone treatment centers. For patients with combined infection or obstruction, the AUA guideline recommends urgent renal drainage within hours. Haas et al. demonstrated that delaying decompression by two or more days was associated with a 29% increase in in-hospital mortality (19). However, rural patients often require long-distance transfer to urban medical centers to receive complex stone surgery, a process that may delay decompression and increase mortality risk (20). Notably, although the West also has vast rural areas, its dense network of high-quality medical resources in coastal cities may partially offset the adverse impact of its rural population, resulting in a relatively slower increase. The timing of the inflection point showed significant spatial heterogeneity: earliest in the Northeast (2014), followed by the West (2015), then the Midwest (2016), and latest in the South (2018). This spatial gradient closely aligns with the geographic distribution patterns of the obesity epidemic and antibiotic resistance reported in previous studies (21, 22) suggesting that the spatiotemporal evolution of these public health indicators may jointly drive the regional disparities in urolithiasis mortality risk. However, additional within-region stratification was not feasible in this study due to small cell counts and CDC WONDER suppression rules. Therefore, the proposed explanations for regional differences remain speculative and should be interpreted as observational associations rather than mechanistic conclusions. Future studies with more granular geographic data and individual-level information are needed to identify the specific drivers of these regional disparities.

Previous studies have generally reported higher urolithiasis mortality in males, consistent with their higher incidence of the disease (23). However, our findings show that female mortality has surpassed that of males, aligning with recent epidemiological reports of a “gender gap reversal” and confirming this trend at the national long-term mortality level for the first time (24). Several factors may contribute to this reversal, reflecting both the evolving epidemiology of stone disease and potential sex-specific mechanisms. First, the incidence of kidney stones among women in the United States has been rising more rapidly than in men over recent decades, and the male-to-female incidence gap has narrowed considerably (25). This shift may be driven by lifestyle changes and increasing obesity rates in women. Second, due to anatomical characteristics, women are more prone to recurrent urinary tract infections and infection-related stones (particularly struvite stones), which are closely associated with the risk of sepsis and may directly increase mortality (11). Third, stone-related symptoms in women are sometimes misdiagnosed as gynecological or gastrointestinal disorders, leading to delays in diagnosis and treatment (26). Additionally, differences in stone composition and metabolic profiles between sexes have been suggested, though the extent to which these contribute to mortality disparities remains unclear. The current dataset does not allow us to distinguish between these potential mechanisms. Future studies with sex-stratified clinical and metabolic data are needed to clarify the specific drivers of this reversal and to inform sex-specific prevention and management strategies. These findings suggest that clinicians should increase vigilance for stone disease in female patients, particularly postmenopausal women with recurrent urinary tract infections, by actively assessing infection control and performing timely surgical intervention when indicated.

Among different racial and ethnic groups, non-Hispanic white people bore the heaviest burden, which may be attributed to their higher prevalence of metabolic syndrome, obesity, and gout—all well-established risk factors for urolithiasis and its severe complications (27). Metabolic syndrome encompasses a cluster of interrelated metabolic abnormalities, including diabetes, hypertension, and hyperlipidemia, which collectively contribute to lithogenic urinary changes. Among these components, diabetes and obesity have been most consistently linked to increased stone formation and complication risks in the literature, and our findings align with these established associations. Hispanic individuals exhibited lower mortality rates compared with non-Hispanic White individuals, despite having a higher burden of metabolic risk factors. Several factors may contribute to this observation. First, geographic distribution may play a role, as Hispanic populations are concentrated in regions with different healthcare infrastructures and mortality coding practices. Second, differential cause-of-death attribution could contribute, as deaths in Hispanic individuals may be more likely to be coded to other causes, leading to an underestimation of urolithiasis-related mortality. Third, true biological or epidemiologic differences, such as lower rates of certain stone types or protective dietary or genetic factors, cannot be excluded. Similar observations have been reported in cardiovascular disease and all-cause mortality (28), but without individual-level data on healthcare access, socioeconomic status, and clinical factors, we cannot distinguish between these possibilities in the current study. Therefore, this finding should be interpreted as an observational association requiring further investigation, rather than a confirmed “paradox.” Non-Hispanic black people had the lowest mortality rate, consistent with previous studies reporting the lowest prevalence of kidney stones in this population (29). This protective effect may be related to higher urinary citrate excretion in Black individuals (30), as citrate is a natural inhibitor of stone formation (31). Notably, both Hispanics and non-Hispanic black people experienced an initial decline in mortality followed by a subsequent increase during the study period. The late-period APC was statistically significant for both groups (2.90% for Hispanics and 6.06% for non-Hispanic black people), indicating that their mortality risk is accelerating and catching up. Although non-Hispanic white people currently bear the heaviest burden, the rising trends observed in Hispanics and non-Hispanic black people during the late period warrant equal attention.

Older adults are at the highest risk of urolithiasis-related mortality. Due to a higher prevalence of chronic diseases, diminished immune function, and poorer tolerance to surgery and anesthesia, they face a significantly higher risk of progressing to sepsis and death once a stone-related infection or obstruction occurs, compared with younger individuals. The 65–74 years age group had the highest full-period AAPC, which may be related to the fact that this age group receives the most active urological surgical interventions. Juliebø-Jones et al. reported that hospital visit rates increased significantly faster among patients aged 70 years and older than among younger populations, with a continuously increasing proportion of surgical interventions (32). This age group is caught in a paradox of “active treatment” versus “high risk"—they actively undergo definitive stone treatment, but their high burden of comorbidities such as hypertension and diabetes exposes them to higher risks of postoperative complications and mortality.

The 75–84 years age group had the highest number of deaths, but a lower mortality rate than those aged 85 years and older, reflecting a “denominator effect” of population age structure: the 75–84 years group has a larger population base, hence the highest absolute death count, whereas the 85 years and older group, despite its smaller population, has a higher mortality rate due to the physiological vulnerability associated with extreme aging. The lack of statistical significance for the APC in the 45–54 years age group during 2018–2024 may be attributable to insufficient statistical power due to the small number of absolute deaths in this age group; nevertheless, the point estimate of 7.08% remains a concerning signal. The late-period APC for those aged 85 years and older reached 11.33%, indicating that mortality risk among the extremely elderly is increasing at an alarming rate.

This study has several strengths. The use of the CDC WONDER database provided population-level, nationally representative mortality data. Joinpoint regression identified significant inflection points in mortality trends. The inclusion of AAMRs reduced differences attributable to population age structure, allowing for more valid comparisons across demographic groups and over time.

However, several limitations must be acknowledged. First, death certificate data are subject to inherent biases. Urolithiasis-related deaths, particularly those with sepsis or renal failure as the immediate cause of death, may be more likely to be attributed to “sepsis” or “chronic kidney disease” rather than “urolithiasis,” leading to systematic underestimation of mortality rates. This is especially relevant for urolithiasis, where death is typically mediated through complications rather than the stone itself, and should be carefully considered when interpreting the observed trends. This is of particular concern when comparing across racial groups and regions. Second, due to the suppression of cells with small counts (<10 cases) in CDC WONDER, mortality data for individuals under 45 years of age were severely incomplete; therefore, this study only included individuals aged 45 years and older, and the findings cannot be extrapolated to younger populations. Third, the database lacks individual-level clinical details, including stone composition, stone burden, specific treatment modalities, postoperative complications, and comorbidity status. Consequently, we were unable to analyze mortality trends by disease severity or treatment approach. Furthermore, the CDC WONDER database does not contain information on treatment modalities or surgical approaches. Over the study period, the management of urolithiasis has shifted substantially from shock wave lithotripsy to endourologic techniques, with broader intervention in older and more comorbid patients. These evolving practices may have influenced perioperative risk and outcomes, but our data cannot distinguish between disease-related and treatment-related mortality. Fourth, this study reports mortality trends without parallel incidence or hospitalization data, making it impossible to determine whether the observed increase reflects a true rise in disease incidence, an increase in case fatality, or changes in death certificate attribution and coding practices over time. Previous studies have reported an increasing prevalence of kidney stones in the U.S. population over past decades (29, 33), but the extent to which this contributes to the mortality trends observed in our study remains speculative. Fifth, this study is descriptive in nature and cannot establish causality. The observed temporal associations between the obesity epidemic, antibiotic resistance, and rising mortality are speculative hypotheses that require further investigation.

## 5 Conclusion

Using the CDC WONDER database, this study analyzed urolithiasis mortality trends among U.S. adults aged 45 years and older from 1999 to 2024. Although mortality increased modestly in the early period, it accelerated dramatically after 2014. At the same time, deep-rooted health disparities persist: female age-adjusted mortality has surpassed that of males, non-Hispanic white people bear the heaviest burden, regional differences are pronounced, and among adults aged 45 years and older, mortality risk increases with age. These findings suggest that urolithiasis mortality has increased substantially since 2014, with notable disparities across regions, sexes, racial groups, and age strata. However, given the low absolute mortality rates and the limitations inherent to death certificate data, these findings should be interpreted as hypothesis-generating. Future studies with clinical data are needed to confirm these trends and to identify the underlying drivers. Future clinical practice and public health interventions may need to focus on high-risk populations (older adults, females, and non-Hispanic white people) and high-risk regions. Of note, the Northeast had the highest AAMR, while the Midwest experienced the fastest increase in mortality; these distinct observations should be considered separately when prioritizing regional interventions. However, given the descriptive nature of this study, these suggestions should be considered hypothesis-generating and require confirmation in future studies. Efforts should aim to narrow disparities and achieve health equity in urolithiasis prevention and control by optimizing access to emergency stone care, strengthening antibiotic management for infection-related stones, and implementing early interventions for metabolic risk factors.

## Data Availability

The original contributions presented in the study are included in the article/Supplementary Material, further inquiries can be directed to the corresponding author.
